# Drugs with meibomian gland expression alone versus combined with intense pulsed light in ocular rosacea: a randomised controlled study

**DOI:** 10.1038/s41433-025-04193-3

**Published:** 2025-12-27

**Authors:** Yuan Gao, Meiting Huang, Wenhui Dong, Yingsi Li, Yu Cheng, Fan Yang, Haili Li, Xiaoming Yan

**Affiliations:** Department of Ophthalmology, Peking University First Hospital, Beijing, China

**Keywords:** Eye diseases, Medical research

## Abstract

**Objectives:**

To determine the effects of intense pulsed light (IPL) combined with meibomian gland expression (MGX) and topical medication on ocular rosacea and assess glutathione peroxidases (GPxs) change in the meibum after IPL treatments.

**Methods:**

Ninety-six eyes of 48 participants with ocular rosacea were randomly assigned to either MGX with erythromycin eye ointment alone (control group) or the combination with IPL (treatment group). During the five visits, participants underwent four IPL or sham IPL sessions at intervals of 2 weeks ± 3 days. The best corrected visual acuity, intraocular pressure, ocular surface disease index (OSDI) questionnaire, lid margin telangiectasia grade, tear break-up time (TBUT), corneal fluorescein staining (CFS), marx line (ML) score, Schirmer’s test, meibomian gland yield secretion score (MGYSS), meibography, *Demodex* were evaluated sequentially. The meibum collected from participants and cryosections of mouse meibomian glands were used for the determination of GPxs or GPx-3.

**Results:**

The TBUT revealed significant improvement in the treatment group compared to the control group (*p* < 0.001). The lower lid margin telangiectasia grade (*p* < 0.001), MGYSS, lower eyelid ML score and CFS exhibited significant improvement in the treatment group compared to the control group. However, the OSDI score, upper lid margin telangiectasia grade, upper eyelid ML score, Schirmer’s test, meibography and *Demodex* showed no statistical difference between the two groups. The GPxs and GPx-3 showed significant increase after IPL treatment.

**Conclusions:**

IPL offered additional benefit in stabilising the tear film, improving the secretion of meibomian gland and reducing abnormal eyelid vascularity. GPxs could be a therapeutic target in IPL treatment.

## Introduction

Meibomian gland dysfunction (MGD) is the most common manifestation among ocular rosacea patients. The morphological abnormalities or telangiectasias of lid margins, occlusive orifices of meibomian gland and the abnormalities of meibum in quality and quantity can be observed in such patients. Besides, conjunctiva, cornea, sclera and uvea can be also involved [1, 2]. Irreversible loss of vision could occur in the severest cases. Thus, it is vital to diagnose and treat ocular rosacea timely and accurately.

There is no universal standard method for the treatment of ocular rosacea. A recent study showed that the combination of intense pulsed light (IPL) and meibomian gland expression (MGX) exhibited a more significant improvement compared with MGX alone [3]. It was also confirmed the efficacy of IPL therapy for ocular rosacea [4]. Moreover, the relief was maintained until nearly 10 months after the IPL routine [4]. Yet, according to the systematic review, there is a lack of high-certainty evidence for the treatment of ocular rosacea [5].

Thus, the study was designed to conduct a rigorous and well-controlled clinical trail to evaluate the efficacy of IPL on ocular rosacea. Meanwhile, the clinical treatment mechanism was preliminarily discussed.

Photobiomodulation is one of the most important molecular biological mechanisms of IPL, which can regulate the reactive oxygen species (ROS) and subsequently leads to the activation or inhibition of a series of signalling pathways downstream [6, 7]. A prior study conducted by our team has already discovered the down-regulation of nicotinamide adenine dinucleotide phosphate oxidase 4 in the IPL-treated mice [8]. Thus, the study aimed to explore whether IPL had antioxidant effects during the treatment process. Glutathione peroxidases (GPxs) are a family of antioxidant enzymes with eight subtypes to down-regulate ROS by catalysing the redox reaction of hydroperoxides [9]. Superoxide dismutases (SODs) are a group of metalloenzymes to defend against oxidative stress. Hence, we specifically centred on the alterations in these two enzymes, which have not been previously documented.

## Materials and methods

### Study design and participants

This investigator-initiated, prospective, randomised, double-blind, paired-eye, sham-controlled trail was conducted at Peking University First Hospital from October 2022 to August 2024. The trail was approved by the Human Research and Ethics Committee of Peking University First Hospital, in accordance with the Declaration of Helsinki, and was registered at http://www.chictr.org.cn (identifier ChiCTR2200063091). Written informed consent was obtained from all subjects before they enrolled.

Participants from the ophthalmology clinic were screened for inclusion and exclusion criteria, subjects aged above 18, clearly diagnosed as rosacea with MGD of grade 2 [10] or above in both eyes, and with a difference in the grade of meibomian gland dysfunction between the eyes of less than 1 grade were recruited. The exclusion criteria were as follows: (1) subjects who had difficulty in follow-up; (2) history of ocular surgery or injury in the past 6 months; (3) laser treatment around the face within 6 months before the study; (4) a history of eye medication other than artificial tears and erythromycin eye ointment in the past 1 month; (5) diagnose of Sjogren’s syndrome, graft-versus-host disease, Stevens-Johnson syndrome, pemphigoid, pregnancy, epilepsy and previous history of facial herpes virus infection; (6) implants or tattoos under the treatment area; (7) wearing contact lenses 48 h before the examination and during the study; (8) taking photosensitive drugs; (9) eyelid abnormalities; (10) history of eye acid or alkali burns.

### Randomisation and masking

The simple randomisation was applied for the two eyes of the eligible subjects to be assigned to undergo IPL and topical erythromycin eye ointment with MGX, or to receive topical erythromycin eye ointment with MGX alone (with sham IPL) at a 1:1 ratio. It was based on the computer-generated sequence created with SPSS 26.0, with allocation concealed using sequentially numbered, opaque, sealed envelopes. The envelopes were kept by the investigator (Dong WH), when the participants would undergo IPL treatment, the investigator would open the envelope and showed the information to the IPL operator (Huang MT). Except for the trial personnel who conducted the IPL treatments, the rest of the parties including the outcome assessor (Gao Y) and participants were kept marked to participant treatment allocation throughout the entire trial. Participant masking was achieved by using a blocking filter (1200 nm) installed on the tip of the IPL probe for the control eye group and the normal IPL probe for the treated eye group. The two probes were identical in appearance.

### Procedures

All participants received four total treatments 2 weeks ± 3 days apart. Prior to each administration of IPL and sham IPL treatment, both groups underwent a session of MGX. A drop of oxybuprocaine hydrochloride eye drops was instilled into the conjunctival sac, followed by massage using a solid stainless-steel tweezer with a flat and expanded pointed end. After this, the IPL treatment was conducted. Prilocaine/lidocaine cream was applied to the treatment region (the forehead and lower eyelid areas from the nasal to the temporal side) for almost 1 h. Subjects were then asked to wash faces, and a layer of ultrasound transmission gel was applied to the treatment region. Two folded pieces of moist gauze were placed on the eyelids with a ceramic patch on the top. The IPL and sham IPL treatments were then administered to the treated eye group and control eye group, respectively, for two sessions. The pulse intensity ranged from 9 to 15 J/cm^2^ hinged on their Fitzpatrick skin type. The number of pulses depended on the length of the treatment region, with each laser spot closely connected to the others, and the overlapping area not exceeding 10%. Participants were required to apply erythromycin ointment to both the upper and lower lid margin once per night during the study period, starting from the first follow-up visit.

### Clinical assessments

Subjects had 5 visits at an interval of 2 weeks ± 3 days, at each visit a comprehensive ophthalmic examination was performed in the following sequence. Best corrected visual acuity (BCVA) and intraocular pressure (IOP) were examined for the adverse events monitor. Ocular surface disease index (OSDI) questionnaire was used to evaluate the subjective symptoms. lid margin telangiectasia grade [11], tear break-up time (TBUT), corneal fluorescein staining (CFS) [12], marx line (ML) score [11] and Schirmer’s Ⅰ test were determined. Meibomian gland yield secretion score [13] (MGYSS, assessment of each of the five glands in the nasal, central and temporal part of eyelids on a scale of zero to three for each gland: 0 = no secretion; 1 = toothpaste-like secretion; 2 = cloudy liquid secretion; 3 = clearly liquid secretion. The total score ranged from 0 to 45 in each upper or lower eyelid). Meibography (SL-D7, TOPCON, JAPAN) which was scored according to a standardised scale: 0 (no loss), 1 (<1/3 loss), 2 (1/3 to 2/3 loss), and 3 (>2/3 loss). The total meiboscore for each eye was calculated by summing the scores of the upper and lower eyelids [13]. The detection of *Demodex folliculorum (D. folliculorum)* from the eyelash was then performed [14]. The ML score, Schirmer’s test, meibography and detection of *Demodex* were performed just in the first and last visits.

### Ocular surface discharge samples

Meibum samples from the treated eyes were collected using swabs during the MGX procedure, while tear fluid samples were obtained using Schirmer strips. All collected samples were immediately stored at −80 °C until further processing.

### GPxs measurement

The total GPxs activity in the meibum (22 samples from 11 participants who were consecutively recruited amid the process) was quantitatively measured using a commercial GPx assay kit (S0059S, Beyotime).

### Animal models

The 11-week-old male Apolipoprotein E (ApoE) knockout mice were obtained from Beijing HFK Bioscience Co, Ltd (Beijing, China). Mice were raised for 13 weeks, fed with normal diet. Next, MGD mice were randomised (unblinded) to either IPL or control group (3 per group-the sample size, the minimum achievable given resource constraints, was deemed sufficient for initial observational assessment). The IPL group received 3-time IPL treatments at an interval of 2 weeks, with no treatments in the control group. Two weeks after the last IPL treatment, all the mice were euthanised to collect lower eyelids. The eyelids were embedded, flash-frozen in liquid nitrogen, and stored at −80 °C. All animal studies were approved by the Experimental Animal Ethics Committee.

### Immunofluorescence

After rewarming, sections were permeabilised (0.1% Triton X-100 in Phosphate-Buffered Saline (PBS), 5 min), blocked (5% Bovine Serum Albumin + 0.1% TritonX-100 in PBS, 1 h at room temperature), and incubated with anti-GPx-3 (1:200, bs-22009R, Bioss) at 4 °C overnight. After PBS washing, sections were incubated with secondary antibody (1:300, ZF-0516, ZSGB-Bio, Beijing, China) at room temperature (90 min in darkness). After PBS washing, Sections were stained with 4′,6-Diamidino-2-Phenylindole (DAPI, ZLI-9557; ZSGB-Bio, Beijing, China), and imaged immediately under a Nikon Eclipse 80i microscope. Image analysis was performed using ImageJ.

### SODs measurement

The total SOD activity in the tear (24 samples from 12 participants) was quantitatively determined using a commercial SOD assay kit (S0101M, Beyotime).

### Statistical analysis

The sample size was calculated assuming a 2-sided α level of 0.05, 80% power, with an expected between-group TBUT difference of 1.8 s, a standard deviation of 3 s after 4 total IPL treatments, and loss to follow-up rate of 10%, 48 participants were required for each group. Statistical analyses were performed using SPSS 26.0 for Windows software (SPSS Inc., Chicago, Illinois, United States). Ranked and categorical data were expressed as frequencies, while quantitative data were expressed as mean differences ± standard deviation or medians and interquartile ranges (IQRs) according to their distribution. The Shaprio–Wilk test was used to evaluate whether the data distribution was normal. As for the comparison of the data from baseline with final follow-up, Paired Samples T test was used for the data corresponded to normal distribution, and Paired Samples Wilcoxon Signed Rank Tests were used for the rest. Statistical significance was based on a two-sided *p* value of less than 0.05.

## Results

**Fig. 1:**
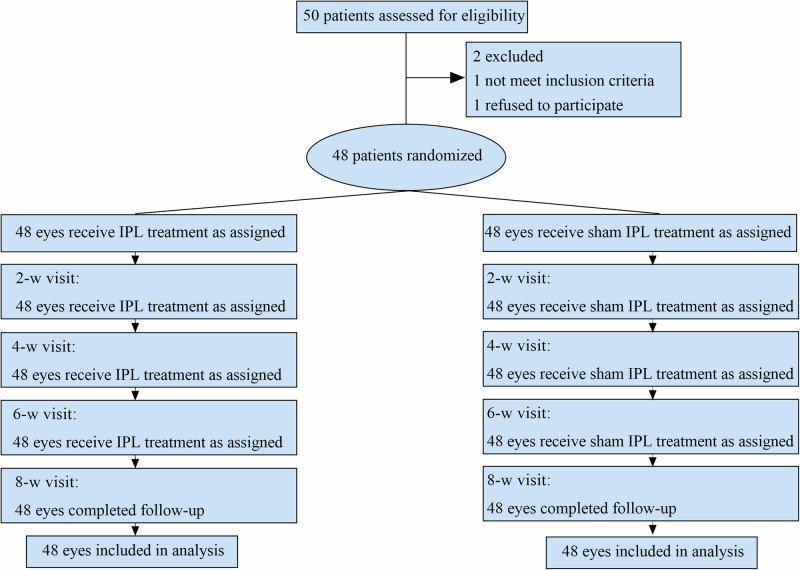
Consolidated standards of reporting trials flow diagram for the study. IPL intense pulsed light.

Figure 1 showed the detail of the trial profile. Forty-eight participants (96%) were enrolled, and their 96 eyes were randomly assigned to either the treatment group or control group. Recruitment stopped once the required sample size was reached. All participants completed the study. Data collected from the 96 eyes of the 48 participants were included in analysis. Throughout the entire trial, no adverse events were reported. The main baseline demographic and clinical characteristics of the participants were well balanced between the trail groups (Table 1).

**Table 1:** Demographic and clinical characteristics of participants in two groups at baseline and final visit.

	IPL-MGX with erythromycin ointment			MGX with erythromycin ointment	
	Baseline		Final visit	Baseline	Final visit
Median age (IQR), years	38 (31–44)				
Gender *n* (%)					
Female	40 (83%)				
Male	8 (17%)				
Median OSDI score (IQR)	20.83 (10.42–37.50)	8.33 (2.08–20.31)		21.88 (10.42–39.06)	10.42 (2.08–21.88)
Upper lid margin telangiectasia grade, *n* (%)					
0	0 (0%)	0 (0%)		0 (0%)	0 (0%)
1	1 (2%)	4 (8%)		5 (10%)	2 (4%)
2	15 (31%)	18 (38%)		12 (25%)	15 (31%)
3	32 (67%)	26 (54%)		31 (65%)	31 (65%)
Lower lid margin telangiectasia grade, *n* (%)					
0	0 (0%)	1 (2%)		0 (0%)	0 (0%)
1	7 (15%)	10 (21%)		7 (15%)	8 (17%)
2	18 (38%)	28 (58%)		20 (42%)	19 (40%)
3	23 (48%)	9 (19%)		21 (44%)	21 (44%)
Median ML score (IQR)					
Upper eyelid (u-ML)	2 (0–5)	2 (0–5)		2(0–5)	2(0–5)
Lower eyelid (l-ML)	3 (0–6)	3 (0–6)		3(0–6)	2(0–6)
Amount of *D. folliculorum*	0 (0–2)	0 (0–0)		0 (0–2)	0 (0–0)
Detection of demodex, *n* (%)					
Positive^a^	8 (17%)	7 (15%)		8 (17%)	6 (13%)
Negative^b^	40 (83%)	41 (85%)		40 (83%)	42 (88%)
Mean MGYSS ± SD					
Upper eyelid (u-MGYSS)	15 ± 5.7	26 ± 8.1		15 ± 6.2	19 ± 7.4
Lower eyelid (l-MGYSS)	16 ± 6.3	26 ± 7.2		17 ± 6.3	20 ± 6.2
Total (t-MGYSS)	31 ± 10.6	52 ± 14.2		32 ± 11.6	39 ± 12.5
Meibography score, *n* (%)					
0	0 (0%)	1 (2%)		0 (0%)	0 (0%)
1	1 (2%)	0 (0%)		0 (0%)	0 (0%)
2	22 (46%)	24 (50%)		23 (48%)	26 (54%)
3	6 (13%)	6 (13%)		5 (10%)	4 (8%)
4	14 (29%)	12 (25%)		13 (27%)	10 (21%)
5	1 (2%)	1 (2%)		3 (6%)	3 (6%)
6	4 (8%)	4 (8%)		4 (8%)	5 (10%)
Median TBUT (IQR), s	3.68 (2.95–4.27)	4.24 (3.63–5.57)		3.64 (3.22–4.31)	3.84 (3.03–4.76)
Median CFS score (IQR)	2 (1–4)	2 (0–3)		2 (0–4)	3 (1–4)
Schirmer’s test value (IQR), mm	15 (7–30)	14 (8–24)		15 (9–25)	14 (9–23)
Schirmer’s test, *n* (%)					
≥10 mm/5 min	32 (67%)	35 (73%)		36 (75%)	35 (73%)
<10 mm/5 min	16 (33%)	13 (27%)		12 (25%)	13 (27%)

*IPL* intense pulsed light, *MGX* meibomian gland expression, *OSDI* ocular surface disease index, *IQR* interquartile range, *ML* Marx line, *MGYSS* meibomian gland yield secretion score, *TBUT* tear break-up time, *CFS* corneal fluorescein staining.

^a^Refers to the presence of ≥3 mites on the 3 lashes of an eyelid.

^b^Refers to the presence of <3 mites on the 3 lashes of an eyelid.

**Fig. 2:**
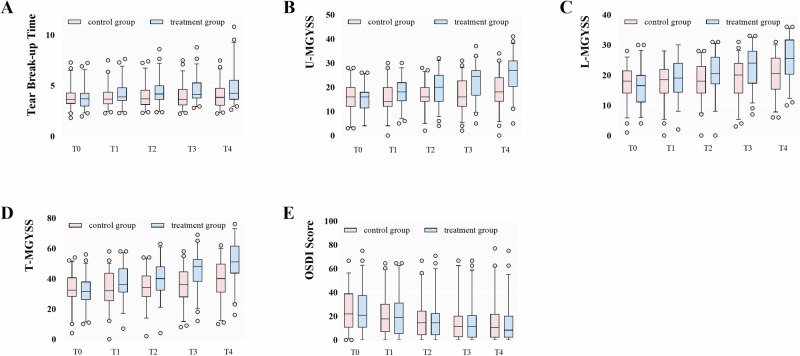
Box plots of raw data at the five visits. TBUT (**A**), u-MGYSS (**B**), l-MGYSS (**C**), t-MGYSS (**D**) and OSDI score (**E**) in the two groups. Data are median (central line), interquartile range (box margins), 5–95 percentile (bottom whiskers end to top whiskers end), and outliers (dots). u-MGYSS upper eyelid meibomian gland yield secretion score, l-MGYSS lower eyelid meibomian gland yield secretion score, t-MGYSS total meibomian gland yield secretion score, OSDI ocular surface disease index.

The TBUT significantly improved from baseline in the treatment group (0.78 s (IQR 0.32–1.47), *p* < 0.001, 95% CI 0.49–1.03) by the last visit (Fig. 2A), while the improvement in the control group was not statistically significant (*p* = 0.22). Moreover, the TBUT significantly increased in the therapy group compared with control group (0.64 s (IQR 0.32–1.27), *p*  < 0.001, 95% CI 0.46–0.83).

**Fig. 3:**
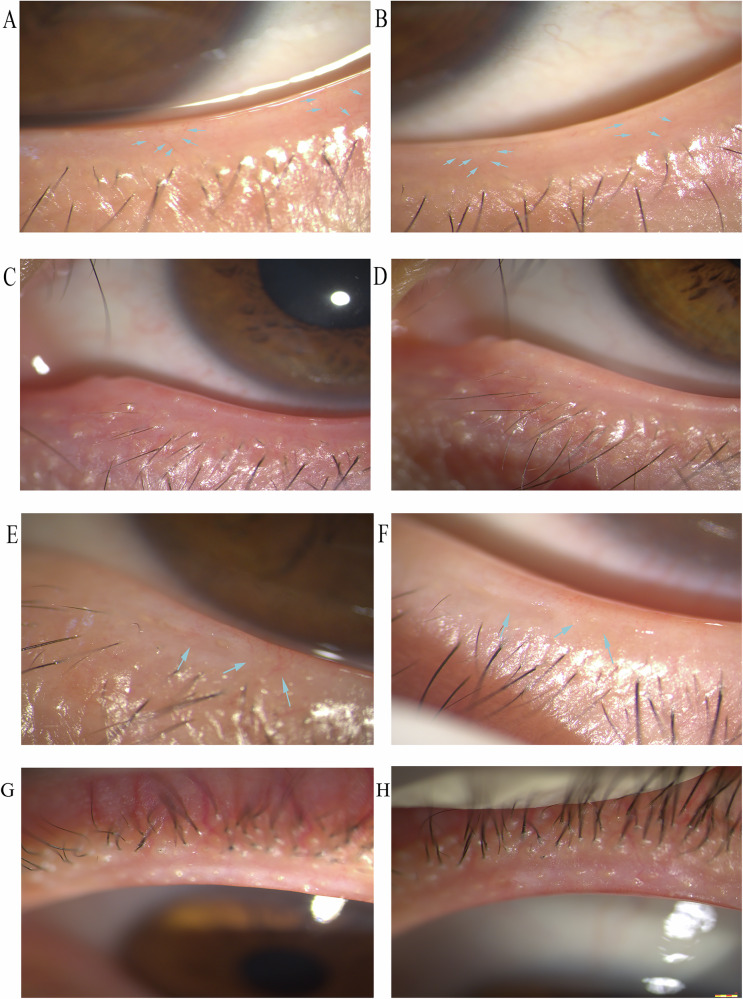
Change of lid margin in the treated eye of four participants. **A**, **B** Patient 1. **A** The dilated capillaries were well-defined at baseline. **B** after four IPL treatments, the abnormal blood vessels got faint, some even regressed. **C**, **D** Patient 2. **C** There was diffuse erythema and oedema of the eyelid, with obstructed meibomian gland orifices at baseline. **D** Marked reduction in eyelid erythema and oedema was observed, with alleviated obstruction of the meibomian gland orifices, and peri-follicular oedema was reduced. **E**, **F** Patient 3. **E** Pre-treatment image showed eyelid margin erythema, notching, and obstructed meibomian gland orifices. **F** Post-treatment image showed resolution of erythema, patency of meibomian gland orifices, and reduction of eyelid margin notching. **G**,**H** Patient 4. **G** Marked plugging of the meibomian gland orifices was present at baseline. **H** Following treatments, the obstruction of the meibomian gland orifices was markedly reduced, allowing for the improved secretion of meibum.

The upper and lower lid margin telangiectasia grade showed significant diminution from baseline to the final visit in the study group (*p* = 0.029; *p* < 0.001), while no significant change was detected in the control group (*p* = 0.41; *p* = 0.76). Figure 3A–F illustrated changes in lid margin telangiectasia in the treated eyes of three participants. By the final visit, the lower lid margin telangiectasia grade showed a significant change between the two groups (*p* < 0.001), yet no significant change was found in the upper lid margin telangiectasia grade (*p* = 0.052).

Analysis of the upper, lower and total MGYSS (u-MGYSS, l-MGYSS and t-MGYSS) showed significant increases from baseline to last follow-up in both groups (*p*  < 0.001 for all, Fig. 2B–D), and the increase was 11 ± 5.7, 9 ± 4.8, 21 ± 8.7 in the study group, respectively, and 4 ± 4.8, 3 ± 4.0, 7 ± 6.9 in the control group, respectively. There was a significant increase from control eyes to treated eyes in the u-MGYSS (7 ± 5.1, *p*  < 0.001), l-MGYSS (5 ± 4.8, *p*  < 0.001), t-MGYSS (12 ± 6.4, *p*  < 0.001), even though the t-MGYSS was higher in the control eyes compared with treated eyes at baseline (2 ± 4.2, *p* = 0.017). Figure 3C–H showed changes in meibomian gland in the treated eyes of three participants.

The OSDI score exhibited a significant decrease from baseline to the final examination in both the treatment group (8.33 (IQR 0.52–20.31), *p*  < 0.001, Fig. 2E) and the control group (8.33 (IQR −1.56–18.75), *p*  < 0.001). However, no statistically significant difference was detected between the two groups by the final examination (*p* = 0.17).

The upper and lower ML score (u-ML, l-ML) showed significant changes from baseline to the final examination in the study group (*p* < 0.001; *p* = 0.001), while no significant change was found in the control group (*p* = 0.77; *p* = 0.29). By the last visit, the l-ML score showed significant change between the two groups (*p* = 0.047), yet no significant change was found in the u-ML score (*p* = 0.14). The study group showed a significant reduction in *D. folliculorum* count from baseline to final examination (*p* = 0.002), though its positive rate did not change significantly within or between groups. No significant intergroup (*p* = 0.27) or intragroup (Study group, *p* = 0.06; Control group, *p* = 0.50) differences were observed in meibography scores. By the final follow-up, the CFS score decreased significantly in the study group compared to the control group (0 (IQR 0–2), *p* = 0.017), though neither group showed significant intragroup change (Study group, *p* = 0.21; Control group, *p* = 0.09). Schirmer’s test value also showed no significant differences between groups (*p* = 0.96) or changes within either group over time (Study group, *p* = 0.67; Control group, *p* = 0.77).

**Fig. 4:**
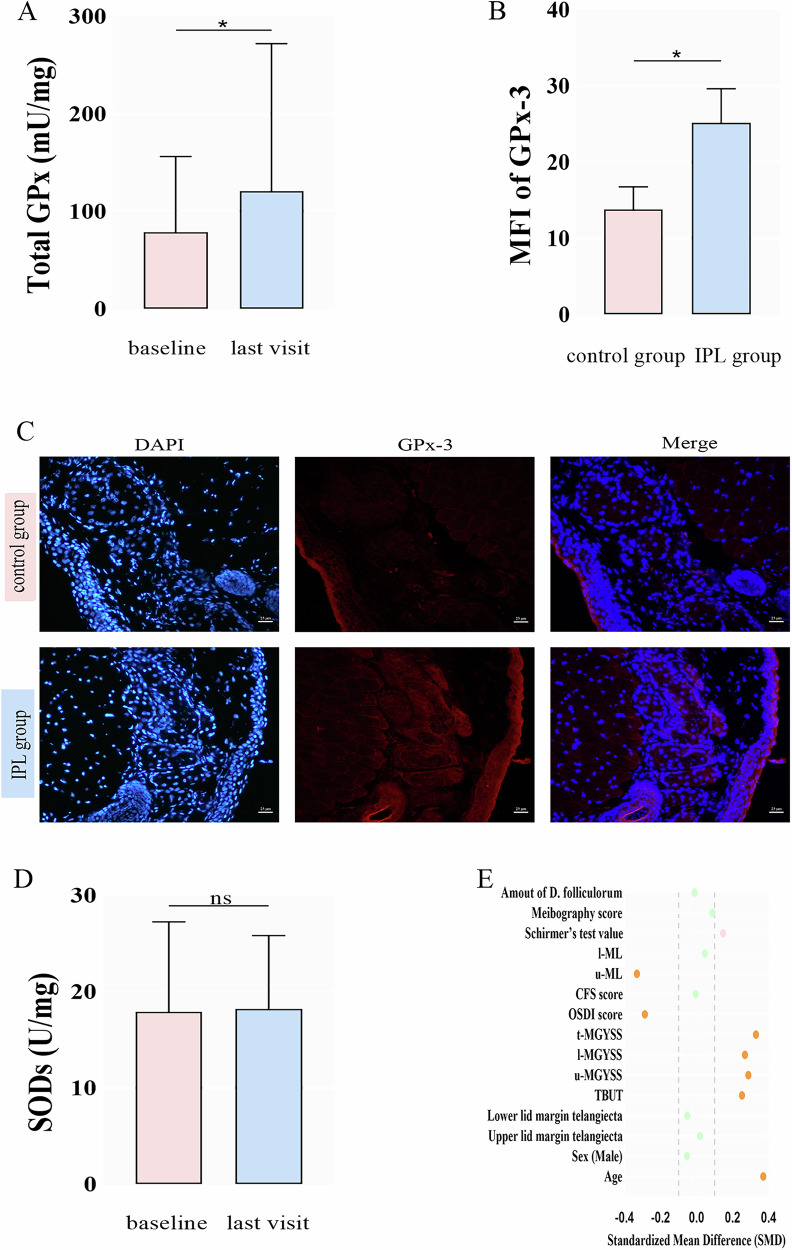
Change of antioxidant enzyme. **A** The concentration of GPxs in the meibum collected from the 11 participants at baseline and last visit. **B** The mean fluorescence intensity (MFI) of GPx-3 in the meibomian gland from the two groups of MGD model mouse. **C** Immunofluorescence microphotograph of meibomian gland from MGD model mouse showing a higher content of GPx-3 (red) in the IPL group compared with the control group. Scale bar represents 25 μm. **D** The concentration of SODs in the tear samples collected from the 12 participants at baseline and last visit. **E** The love plot assessing the representativeness of the meibum collection Subgroup. TBUT, tear break-up time; u-MGYSS, l-MGYSS and t-MGYSS, upper, lower and total eyelid meibomian gland yield secretion score; OSDI, ocular surface disease index; CFS, corneal fluorescein staining; u-ML, l-ML, upper and lower marx line.

There was a significant increase from baseline to the final examination in the concentration of total GPx in the meibum of the treated eyes (187.12 ± 280.44, 272.34 ± 339.98 mU/mg protein, *p* = 0.028, Fig. 4A). The mean fluorescence intensity of GPx-3 in the frozen sections from MGD model mouse also showed significant increase in the IPL group compared with the control group (25.16 ± 4.43, 14.65 ± 1.84, *p* = 0.019, Fig. 4B, C). There was no statistically significant change in tear SODs concentrations from baseline to final examination (19.99 ± 9.81, 19.45 ± 7.58 U/mg protein, Fig. 4D).

Baseline demographic and clinical characteristics were well-balanced between the meibum-sampled subgroup and the remaining patients (Fig. 4E). Key clinical outcomes (e.g., TBUT, OSDI score, MGYSS) in the subgroup showed statistically significant improvement from baseline to the final visit (*p* < 0.01 for all).

## Discussion

The therapeutic efficacy of IPL for ocular rosacea remains an area of ongoing investigation and uncertainty. To our knowledge, this is the first prospective randomised controlled study to report the efficacy of topical agents with MGX alone and in combination with IPL in ocular rosacea. The study demonstrated that IPL-MGX in addition to erythromycin ointment resulted in improved indicators relevant to DED compared with MGX with erythromycin ointment alone in participants presenting with MGD due to ocular rosacea.

The TBUT showed a significant increase in the study group, indicating that IPL can help maintain or improve tear film stability, in agreement with other research [4]. Nevertheless, the increase in TBUT was only 0.64 s (IQR 0.32–1.27) from baseline and 0.78 s (IQR 0.32–1.47) compared to the control group. These increments were smaller than the increase (2.18 ± 2.54 s) reported in other study [15]. It is possible that the lower increase of TBUT in our study can be attributed to a shorter TBUT at baseline (3.68 s), while the baseline data in their study were 7.64 ± 2.23 s. Patients with short TBUT-type dry eye [16] are characterised by TBUT <5 s along with obvious subjective symptoms. The abnormality of mucin and lipid can induce short TBUT-type dry eye, and the mucin layer even plays a more important role in the pathogenesis [17]. However, a study showed that IPL had no effect on the expression of mucin MUC5AC [18] and Caballero et al. [19] found that IPL could not benefit to mucin layer. Among the participants in our study, the majority had short TBUT-type dry eye, making it difficult to improve TBUT. Since the mechanism of IPL’s effect on TBUT is based on its anti-inflammatory effects and its effect on the meibomian gland rather than its effect on the mucin layer.

The upper eyelid margin telangiectasia grade showed no significant change between the two groups, whereas the lower eyelid margin telangiectasia grade differed significantly. This discrepancy may be attributed to the differing distances from the upper versus lower eyelid to the IPL probe. A greater distance could lead to higher luminous energy loss, as light may be absorbed by melanin and haemoglobin in non-target tissues. The increase of MGYSS were significantly greater in the IPL group, indicating that the IPL can provide additional benefits in the improvement of MGD.

In our study, the OSDI score showed a significant decrease in the IPL group, indicating that the subjective symptoms had declined. However, there was no evidence to support that IPL could bring extra benefits in symptomatic improvements, as the difference between the IPL group and control group was not statistically significant. This result differed from the other study, which found the symptomatic improvements were greater in the IPL-MGX group compared with MGX group [3]. This inconsistency might be due to our different study design, as we conducted a paired-eye self-control study. Therefore, it may have been difficult for some participants to distinguish the symptoms between the two eyes.

No significant change was found in meibography scores, consistent with prior reports [3], likely because IPL cannot reverse complete gland atrophy and may require longer treatment to improve partial atrophy. The lack of CFS reduction may be due to low baseline scores (>60% of participants had CFS < 2). Similarly, Schirmer’s test results did not improve significantly, aligning with the inconsistent literature on IPL’s effect on tear secretion.

So far, few previous studies on the effects of IPL treatment on antioxidant enzymes activity in ocular surface, including its effects on GPxs, have been found. Thus, the present pioneering study reveals that the antioxidant enzymes especially GPxs might be a therapeutic target on the IPL treatment for ocular rosacea. Our pilot study showed that the concentration of GPx-3 in the meibum increased significantly after the IPL treatment. Subsequently, we employed immunofluorescence and enzyme-linked immunosorbent assay (ELISA) techniques to validate the experimental results. GPx-3 prevail in adipose tissue may limit the accumulation of ROS to regulate oxidative stress [20]. The increase of the concentration of total GPx or GPx-3 prompted that IPL might be able to elevate the activity of antioxidant enzymes. A study found that IPL could preserve the activity of superoxide dismutase (SOD) and catalase (CAT) in the skin cells [21]. However, the evidence that support IPL can up-regulate SOD does not be found in our study.

There are some limitations in our study. First, we lack concurrent untreated controls for GPX analysis in the human trial and terminal design precluded baseline GPX measurement in the mouse study. Thus, the evidence for GPX up-regulation, while statistically significant, is preliminary. Future studies with paired control samples are needed for confirmation. Second, the paired-eye design limits the interpretation of subjective outcomes, as patients cannot reliably differentiate symptoms between eyes. Lastly, the majority of participants had TBUT shorter than 5 s, similar studies included subjects with a range of TBUT values will be required.

Overall, this prospective randomised trial indicated that IPL-MGX in addition to erythromycin ointment was associated with improved stability of tear film and function of meibomian gland compared to MGX with erythromycin ointment alone, without safety concerns. Besides, our study support that IPL can promote the activity of GPxs in the meibomian gland.

## Summary

### What was known before

- IPL may be an effective treatment for ocular rosacea.

### What this study adds

- IPL could be beneficial to ocular rosacea.
- IPL could affect the antioxidant enzyme in the ocular surface.
- GPxs could be a therapeutic target in IPL treatment.

## Data Availability

The data that support the findings of this study are available from the corresponding author upon reasonable request.

## References

[1] Ghanem VC, Mehra N, Wong S, Mannis MJ. The prevalence of ocular signs in acne rosacea. Cornea. 2003;22:230–3. 12658088 10.1097/00003226-200304000-00009

[2] Redd TK, Seitzman GD. Ocular rosacea. Curre Opin Ophthalmol. 2020;31:503–7. 10.1097/ICU.0000000000000706 33009083

[3] Sagaser S, Butterfield R, Kosiorek H, Kusne Y, Maldonado J, Fautsch MP, et al. Effects of intense pulsed light on tear film TGF-β and microbiome in ocular rosacea with dry eye. Clinical Ophthalmol. 2021;15:323–30. 10.2147/OPTH.S280707 PMC7850425 33536740

[4] Seo KY, Kang SM, Ha DY, Chin HS, Jung JW. Long-term effects of intense pulsed light treatment on the ocular surface in patients with rosacea-associated meibomian gland dysfunction. Contact Lens Anterio. 2018;41:430–5. 10.1016/j.clae.2018.06.002 29958778

[5] Zuuren EJ, Fedorowicz Z, Tan J, Linden MMD, Arents BWM, Carter B, et al. Interventions for rosacea based on the phenotype approach: an updated systematic review includingGRADEassessments. Brit J Dermatol. 2019;181:65–79. 30585305 10.1111/bjd.17590 PMC6850438

[6] Zeng HY, Gong L. A review of applications and intracellular mechanisms of intense pulsed light in eyelid inflammatory diseases. Photobiomod Photomed. 2023;41:104–19. 10.1089/photob.2022.0120 36927050

[7] Murphy MP. How mitochondria produce reactive oxygen species. Biochem J. 2009;417:1–13. 19061483 10.1042/BJ20081386 PMC2605959

[8] Xie LY, Song WJ, Dong WH, Li YS, Chen SD, Sun XA, et al. Indirect application of intense pulsed light induces therapeutic effects on experimental murine meibomian gland dysfunction. Front Med-Lausanne. 2022;9:923280. 35721080 10.3389/fmed.2022.923280 PMC9201038

[9] Brigelius-Flohé R, Flohé L. Regulatory Phenomena in the Glutathione Peroxidase Superfamily. Antioxidants Redox Signal. 2020;33:498–516. 10.1089/ars.2019.7905 31822117

[10] Geerling G, Tauber J, Baudouin C, Goto E, Matsumoto Y, O'Brien T, et al. The international workshop on meibomian gland dysfunction: report of the subcommittee on management and treatment of meibomian gland dysfunction. Invest Ophthalmol Vis Sci. 2011;52:2050–64. 21450919 10.1167/iovs.10-6997g PMC3072163

[11] Arita R, Minoura I, Morishige N, Shirakawa R, Fukuoka S, Asai K, et al. Development of definitive and reliable grading scales for meibomian gland dysfunction. Am J Ophthalmol. 2016;169:125–37. 27345733 10.1016/j.ajo.2016.06.025

[12] Association CGoOBoCM. Experts’ consensus about clinical diagnosis and treatment of dry eye (2013). Chin J Ophthalmol. 2013;49:73–5.

[13] Society CBotADE, Association OSaTFDGoOCoC-SME, Association OSaDEGoCO. Chinese expert consensus on meibomian gland dysfunction: diagnosis and management. Chin J Ophthalmol. 2023;2023:880–7. 10.3760/cma.j.cn112142-20230822-00054 37936356

[14] Society CBotADE, Association OSaTFDGoOCoC-SME. Chinese expert consensus on diagnosis and treatment of Demodex blepharitis. Chin J Ophthalmol. 2018;54:491–5.

[15] Rong B, Tang Y, Liu RX, Tu P, Qiao J, Song WJ, et al. Long-term effects of intense pulsed light combined with meibomian gland expression in the treatment of meibomian gland dysfunction. Photomed Laser Surg. 2018;36:562–7. 30251914 10.1089/pho.2018.4499

[16] Toda I, Shimazaki J, Tsubota K. Dry eye with only decreased tear break-up time is sometimes associated with allergic conjunctivitis. Ophthalmology. 1995;102:302–9. 7862418 10.1016/s0161-6420(95)31024-x

[17] Tsubota K. Short tear film breakup time-type dry eye. Invest Ophth Vis Sci. 2018;59:DES64–70. 10.1167/iovs.17-23746 30481808

[18] Xue AL, Wang MTM, Ormonde SE, Craig JP. Randomised double-masked placebo-controlled trial of the cumulative treatment efficacy profile of intense pulsed light therapy for meibomian gland dysfunction. Ocul Surf. 2020;18:286–97. 32007523 10.1016/j.jtos.2020.01.003

[19] Guilloto Caballero S, García Madrona JL, Colmenero Reina E. Efecto del láser de luz pulsada en pacientes con síndrome de ojo seco. Archivos de la Sociedad Española de Oftalmología. 2017;92:509–15. 28256362 10.1016/j.oftal.2016.12.018

[20] Song YJ, Zhu MJ, Islam MA, Gu WY, Alim K, Cheng CS, et al. Glutathione peroxidase 3 is essential for countering senescence in adipose remodelling by maintaining mitochondrial homeostasis. Redox Biol. 2024;77:103365. 39312866 10.1016/j.redox.2024.103365 PMC11447410

[21] Kim J, Lee J, Choi H. Intense pulsed light attenuates UV-induced hyperimmune response and pigmentation in human skin cells. Int J Mol Sci. 2021;22:3173. 33804685 10.3390/ijms22063173 PMC8003787

